# Cryptanalysis and Improvement of Several Identity-Based Authenticated and Pairing-Free Key Agreement Protocols for IoT Applications

**DOI:** 10.3390/s24010061

**Published:** 2023-12-22

**Authors:** Haiyan Sun, Chaoyang Li, Jianwei Zhang, Shujun Liang, Wanwei Huang

**Affiliations:** College of Software Engineering, Zhengzhou University of Light Industry, Zhengzhou 450001, China; sunhaiyan@zzuli.edu.cn (H.S.);

**Keywords:** AKA, identity-based cryptography, eCK security model, attacks

## Abstract

Internet of Things (IoT) applications have been increasingly developed. Authenticated key agreement (AKA) plays an essential role in secure communication in IoT applications. Without the PKI certificate and high time-complexity bilinear pairing operations, identity-based AKA (ID-AKA) protocols without pairings are more suitable for protecting the keys in IoT applications. In recent years, many pairing-free ID-AKA protocols have been proposed. Moreover, these protocols have some security flaws or relatively extensive computation and communication efficiency. Focusing on these problems, the security analyses of some recently proposed protocols have been provided first. We then proposed a family of eCK secure ID-AKA protocols without pairings to solve these security problems, which can be applied in IoT applications to guarantee communication security. Meanwhile, the security proofs of these proposed ID-AKA protocols are provided, which show they can hold provable eCK security. Some more efficient instantiations have been provided, which show the efficient performance of these proposed ID-AKA protocols. Moreover, comparisons with similar schemes have shown that these protocols have the least computation and communication efficiency at the same time.

## 1. Introduction

Internet of Things (IoT) applications have been increasingly exploited as information technology, operational technology, communication technology, and electronic technology develop. Examples include Smart Grid, Internet of Vehicles, Industrial IoT, and Agriculture IoT, which have enhanced living conditions greatly. With the development of 5G, secure and efficient communication demands have become huge as massive IoT devices are participating in these IoT applications. In general, IoT end devices and IoT servers play important roles in IoT applications. IoT end devices, mounted with sensors, collect useful information and transmit data to the IoT servers over the open network. When these IoT data are transmitted among different IoT devices, the sensitive information inserted in these IoT data needs more secure cryptographic algorithms to protect IoT user privacy and data security.

To protect the security of data transmission, cryptographic means that require secret keys are presented. For IoT user identity authentication, the shared key, public key certificate, and zero-knowledge proof are some common cryptographic methods [1]. For secure IoT data transmission, the digital signature can help confirm data ownership [2], the key agreement can help establish a secure session key [3], and the public key encryption can guarantee IoT data security with ciphertext in the public Internet environment [4]. For IoT data with fine-grained access control, attribute-based encryption can help establish a secure access control strategy with the attributes of IoT users and data [5], and security policy protocol can solve Internet control message protocol (ICMP) attacks [6]. Many cryptographic algorithms guarantee secure and efficient communication among different IoT applications—there are too many to mention one by one. However, these cryptographic algorithms are public; therefore, the keys are essential for secure data transmission. Moreover, blockchain technology has been applied to IoT applications to solve the centralized management problem [7].

Key agreement (KA) is an important method to protect keys, which supports building a common session key between no less than two different users for subsequent communications. Authenticated key agreement (AKA) not only generates a common session key, but also prevents active attacks and implicitly authenticates the participants simultaneously. In the public-key infrastructure (PKI) setting, the user’s long-term public key is matched with the corresponding identity in a certificate, which is derived by a trusted certificate authority. However, managing and transmitting these certificates results in heavy computation and storage costs. Considering IoT end-devices are usually resource-constrained and have limited memory, computation power, storage, and battery life, PKI-based AKA protocols (e.g., [3,8]) are not suitable for IoT applications. Therefore, to avoid the PKI certificate problem, AKA (ID-AKA) protocols with identity are proposed [9]. In ID-AKA protocols, every user owns a unique identity, the long-term public key is constructed by the user’s personal identity, and the private key is composed of the identity and a master key, which is created by the trusted key generation center (KGC). Since the introduction of the first ID-AKA protocol in [10], many ID-AKA protocols have been introduced based on bilinear pairings [11,12,13,14,15,16,17,18], which is a high time-complexity operation. Because of the resource restriction of IoT end devices, ID-AKA protocols without pairings are more suitable for IoT applications.

Currently, the modified Bellare and Rogaway (mBR) model [12], the Canetti–Krawczyk (CK) model [19], and the extended Canetti–Krawczyk (eCK) model [20] are some famous security models for AKA protocols. In particular, the eCK model can achieve the most security properties [13]. Meanwhile, to satisfy the communication demands of the high speed, low latency, and large connections in IoT applications, a more secure and efficient AKA protocol is needed. Thus, designing efficient ID-AKA protocols without pairings while maintaining eCK security would be more suitable for IoT applications.

### Motivation and Our Contribution

Aiming at considering the aforementioned problems, this paper presents some efficient ID-AKA protocols while holding the eCK security for IoT applications.

- We provide the preliminaries of some Diffie–Hellman assumptions and the forking lemma, provide a detailed description of the eCK-security model for two-party ID-AKA protocols, and draw a figure to show the network model for these protocols.
- We analyze three recently proposed ID-AKA protocols without pairings [21,22,23], and point out the security flaws against some known attacks.
- We propose a family of pairing-free ID-AKA protocols in the eCK security model. The security proof also considers the case where the public key materials related to the long-term private key can be altered by an active adversary. Furthermore, events in our security proof are complementary.
- We provide some more efficient protocols that need four elliptic curve point multiplication operations. Protocol comparison shows that our efficient protocols have the advantage over similar protocols with the items of security, computation, and communication efficiency.

The paper is organized as follows: Section 2 provides some related work, Section 3 presents the cryptanalysis of several ID-AKA protocols, Section 4 proposes a family of pairing-free ID-AKA protocols, Section 5 shows some more efficient instantiations, Section 6 presents the performance and comparison, and Section 7 gives the conclusion.

## 2. Related Work

Some related works about the cryptographic methods for IoT applications and developments of KA protocols are provided in the following two subsections.

### 2.1. Cryptographic Methods for IoT Applications

In IoT applications, the IoT data generally contain plenty of sensitive information about the system users, such as their personal identity, location, and production data. When these IoT data are transmitted among different IoT devices and applications, cryptographic technologies play an essential role in the processes of user identity authentication, IoT data transmission, IoT data fine-grained access control, and so on. Jayabalasamy et al. [2] proposed an aggregate signature scheme to solve the nonrepudiation problem in blockchain ecosystems, which could also confirm the data ownership in the untrusted IoT environment. Li et al. [3] designed a KA protocol based on the SM2 algorithm, which could help establish a secure session key between different system users in smart grid communications. Pu et al. [4] introduced a public key authentication encryption scheme, and added the function of keyword search into this scheme to guarantee IoT data security in industrial IoT applications. Rasori et al. [5] provided a survey of the attributed-based encryption protocols in recent years, and figured out the problems and challenges of IoT data fine-grained access control in most current IoT applications. Onyema et al. [6] presented a security policy protocol for the detection and prevention of Internet control message protocol (ICMP) attacks in software-defined networks. For these different cryptographic algorithms, the key is an essential part of secure IoT communication. Therefore, establishing a secure session key is the first issue that should be taken into consideration for secure communication in IoT applications.

### 2.2. Developments of KA Protocols

Numerous interesting pairing-free ID-AKA protocols have been introduced in recent years. In 2010, Cao et al. [24] provided a one-round ID-AKA protocol without pairing and presented the security proof in the mBR model. Then, two pairing-free ID-AKA protocols were proposed in [25,26], which utilized the CK model to prove its security. In 2016, Ni et al. [27] showed that the security proof of Sun et al.’s protocol neglected the case where the public key materials related to the long-term private key could be altered by an active adversary, and proposed two eCK-secure ID-AKA protocols without pairings. However, events in the security proof of their protocols were not complementary, which resulted in a mismatch in the freshness definition. Moreover, KGC needed to generate one extra long-term private key, which increased the storage, computation, and communication costs with the increase in the number of users. Bala et al. [21] presented an ID-AKA scheme without pairing for wireless sensor networks and claimed it was secure in the eCK security model. But Dang et al. [28] showed that its security proof had the same problem. Furthermore, the eCK security model was not secure as it suffered from an ephemeral key reveal attack.

In 2018, Dang et al. [28] provided a pairing-free ID-AKA protocol that could achieve eCK security in vehicular ad hoc networks. However, in 2021, Deng et al. [29] showed that it was not eCK-secure and put forward a new scheme that required only four scale multiplication operations. However, we found that the security proof had some flaws, for example, it was inappropriate that the challenger grasped the private key of KGC in the proof Cases CA2, CA3, CA4, and CA6. Mohammadali et al. [22] proposed the NIKE protocol, an ID-AKA protocol without pairing. However, there were still some drawbacks to it. Firstly, we found this scheme had a design flaw, i.e., if an individual was to learn about the long-term private keys of two parties (say A(Meter) and B(AHE)), they could easily obtain KGC’s master key. Secondly, it could not resist a KCI attack. Thirdly, although the NIKE protocol only needed, at most, three elliptic curve point multiplication operations, it needed three hash-to-point operations, which was a more time-consuming operation than the point multiplication operation. In 2019, Zhang et al. [23] gave two pairing-free and unbalanced ID-AKA protocols for disaster scenarios. Their protocols were actually unbalanced versions of the protocol in [24]. Their protocols reduced one elliptic curve point multiplication operation for the limited party. However, Zhang et al.’s protocols did not have ephemeral key reveal resistance. In 2020, Daniel et al. [30] pointed out that Bala et al.’s protocol [21] could not resist an ephemeral key reveal attack. They also provided an ID-AKA protocol and presented its security proof in the eCK model [27]. However, its computational cost was still higher, which needed five-point multiplication operations in the elliptic curve. Furthermore, they pointed out that protocol [27] suffered from key offset attacks; however, key offset attacks could be simply avoided by adding a message authentication code using the same method in [30].

In 2021, Kumar and Chand [31] presented an ID-AKA protocol with cloud for the wireless body area network for anonymous health data authentication. However, Rakeei and Moazami [32] pointed out that this protocol could not resist a man-in-the-middle attack and achieve perfect forward secrecy. In 2022, Pu et al. [33] provided a mutual authentication and KA protocol for data privacy preserving in unmanned aerial vehicles (UAVs). Zhang et al. [34] designed a group key agreement (GKA) protocol for user privacy protection and data resource secure sharing in an intelligent IoT system. In 2023, Zhou et al. [35] presented an AGKA protocol for an AI-based automation system, which utilized a semi-trusted authority to perform precomputation operations. Pan et al. [36] focused on the communication security of UAVs to introduce a heterogeneous AKA protocol. Zhang et al. [37] provided a symmetric-key AKA protocol for edge-cloud IIoT, which could achieve perfect forward secrecy based on both authentication and derivation master keys. Abdussami et al. [38] proposed an AKA protocol for secure patient health-related data sharing in IoMT.

The security comparisons of these ID-AKA protocols are shown in Table 1, and the security items of the KGC’s master key (MSS), weak perfect forward secrecy (wPFS), key compromise impersonation resilience (KCIR), ephemeral secrets reveal resistance (ESRR), and assumption (AS) were compared. Facing these problems and secure IoT communication demands, a secure ID-AKA protocol is needed to strengthen the security of session keys between different IoT users. The next sections will present the cryptanalysis of several ID-AKA protocols [21,22,23] first, and then provide the proposed ID-AKA protocols.

## 3. Preliminaries

Some basic concepts including complexity assumptions and the eCK security model for two-party ID-AKA protocols are reviewed in this section.

### 3.1. Complexity Assumptions

Let G be an elliptic curve additive group with a large prime order *q*, and *P* is a generator of G. Some Diffie–Hellman assumptions over G are recalled as follows.

- **Computational Diffie–Hellman (CDH) Assumption**: Given three points, P,aP, and bP, where $a,b\in Z_{q}^{*}$, the advantage $Adv_{M}^{CDH}$ to compute abP is negligible for any probabilistic polynomial time (PPT) adversary M.
- **Decision Diffie–Hellman (DDH) Assumption**: Given four points P,aP,bP, and cP, where $a,b,c\in Z_{q}^{*}$, the advantage $Adv_{M}^{DDH}$ to decide whether c≡ab mod *q* is negligible for any PPT adversary M.
- **Gap Diffie–Hellman (GDH) Assumption**: Given four points P,aP,bP, and cP, where $a,b,c\in Z_{q}^{*}$, the advantage $Adv_{M}^{GDH}$ to compute abP by accessing a DDH oracle is negligible for any PPT adversary M.

### 3.2. The Forking Lemma

The forking lemma is applied in the security proof of our proposed protocols. Here, we recall it described in [27].

Let $\Theta_{S}$ be a generic digital signature scheme. Given an input message *m*, $\Theta_{S}$ produces a triple (K,h,v), where *K* is a randomly selected value in a large set, *h* is the hash value of (m,K), and *v* is only dependent on *K*, *m*, and *h*. Assume that a PPT algorithm M can produce a valid signature (K,h,v) on the message *m* with non-negligible probability. Then, with non-negligible probability, a replay of this algorithm can output two valid signatures (K,h,v) and $\left(K,\bar{h},\bar{v}\right)$ on the same message *m*, such that $h\neq \bar{h}$.

### 3.3. eCK-Security Model for Two-Party ID-AKA Protocols

We now recall the eCK-security model for two-party ID-AKA protocols in [13,27].

- **Participants.** Let $U=\{ID_{1},\cdots ,ID_{L}\}$ be a finite set of *L* honest parties. Each participant $ID_{i}\in U$ is modeled as a PPT Turing machine. Any two parties can be involved in a protocol execution. Each party may execute multiple instances (sessions) in parallel. Let $\prod_{i,j}^{m}$ denote the *m*th protocol session, which runs at party $ID_{i}$ (the owner) with intended partner party $ID_{j}$. Every session $\prod_{i,j}^{m}$ has internal state variables $State_{i,j}^{m}$ and $tran_{i,j}^{m}$ to record the state of $\prod_{i,j}^{m}$, and the transcript of messages sent and received by $\prod_{i,j}^{m}$, respectively. If $\prod_{i,j}^{m}$ can compute a session key $SK_{i,j}^{m}$, $State_{i,j}^{m}=completed$. The messages in $tran_{i,j}^{m}$ are ordered according to the protocol specification.
- **Adversary Model.** The adversary M is modeled as a PPT Turing machine and has full control of the communication network. Active attacks are formulated by allowing the adversary M to perform the following queries:
  – EphemeralKeyReveal ($\prod_{i,j}^{m}$). The adversary M is provided the ephemeral private key of $\prod_{i,j}^{m}$.
  – SessionKeyReveal ($\prod_{i,j}^{m}$). The session key held by a completed session $\prod_{i,j}^{m}$ is returned to M.
  – Corrupt ($ID_{i}$). The long-term private key of $ID_{i}$ is returned to the adversary M.
  – KGCStaticKeyReveal. The adversary M obtains the master key of KGC. This query is used to model master key forward secrecy.
  – RegCT ($ID_{i}$). Via this query, the adversary M is able to register a dishonest party with identity $ID_{i}$. Meanwhile, M obtains $ID_{i}$’s long-term private key and totally controls $ID_{i}$.
  – Send ($\prod_{i,j}^{m},M$). Via this query, the adversary M can send any message *M* to party $ID_{i}$ in session $\prod_{i,j}^{m}$ on behalf of party $ID_{j}$. The adversary M is responded to according to the protocol specification. $\prod_{i,j}^{m}$ can be initiated by $ID_{i}$ when M=λ. In general, for simplicity $ID_{i}\neq ID_{j}$ is required, i.e., two identical participants will not run a session. Internal states of $\prod_{i,j}^{m}$ should be maintained accordingly.
  – **Test ($\prod_{i,j}^{m}$)**. The input session $\prod_{i,j}^{m}$ must be fresh. In response to this query, $\prod_{i,j}^{m}$ flips a fair coin b∈{0,1}, and returns the real session key if b=0, or a random sample from the distribution of the session key if b=1.
- **Security Experiment.** The security experiment between the adversary M and the challenger CH consists of the following phases.
  – **Setup.** The challenger CH generates the system parameters along with the master private key and valid long-term secret keys for each party. The adversary M is then provided all public data, including the identities of all the honest parties.
  – **The first phase of the game.** Adversary M is allowed to issue a polynomial number of EphemeralKeyReveal, SessionKeyReveal, Corrupt, KGCStaticKeyReveal, RegCT, and Send queries in any order.
  – **The second phase of the game.** At some point, adversary M chooses a fresh session $\prod_{i,j}^{m}$ (see Definition 2) and issues a Test($\prod_{i,j}^{m}$) query at most once. After this, adversary M can keep asking other queries under the condition that the test session must remain fresh.
  – **The end of the game.**M makes a guess $b^{\prime }$ for *b*.
- **Advantage.**M wins the above security experiment if the test session $\prod_{i,j}^{m}$ is still fresh and $b^{\prime }=b$. The advantage of M in winning the above security experiment is defined as $Adv^{AKE}\left(M\right)=\left|2Pr\left[M\;wins\right]-1\right|$, where M wins refers to M can distinguish the tested session key from a random string.

In the following, we introduce definitions for Matching Session, Freshness, and eCK Security.

**Definition** **1** (Matching Session)**.** *If two completed sessions $\prod_{i,j}^{m}$ and $\prod_{j,i}^{n}$ have the same message transcript, they are said to be matching.*

**Definition** **2** (Freshness)**.** *Let* $\prod_{i,j}^{m}$ *be a completed session between honest party* $ID_{i}$ *and* $ID_{j}$, $\prod_{i,j}^{m}$ *is said to be fresh if none of the following three conditions hold:*

*(1)*  *The adversary M knows the session key of $\prod_{i,j}^{m}$ or its matching session $\prod_{j,i}^{n}$ (if $\prod_{j,i}^{n}$ exists);*
*(2)*  *$\prod_{i,j}^{m}$ has a matching session $\prod_{j,i}^{n}$, and the adversary M knows both the long-term private key of participant $ID_{i}$ and the ephemeral private key of $\prod_{i,j}^{m}$, or both the long-term private key of participant $ID_{j}$ and the ephemeral private key of $\prod_{j,i}^{n}$.*
*(3)*  *$\prod_{i,j}^{m}$ has no matching session, and the adversary M knows both the long-term private key of participant $ID_{i}$ and the ephemeral private key of $\prod_{i,j}^{m}$, or the long-term private key of participant $ID_{j}$.*

**Remark** **1.** *The first condition in Definition 2 is to exclude the trivial attack that M obtains the session key directly. The second and third conditions in Definition 2 are to exclude the trivial attack that M obtains the long-term private key and the ephemeral private key of one party simultaneously. If $\prod_{i,j}^{m}$ has no matching session, it means that M has obtained the ephemeral private key of participant $ID_{j}$; therefore, M cannot obtain the long-term private key of $ID_{j}$.*

**Definition** **3** (eCK Security)**.** *We say that an ID-AKA protocol is secure in the eCK model if the following conditions hold:*

*(1)*  *If two honest parties successfully complete matching sessions, they both compute the same session key.*
*(2)*  *For any PPT adversary,*M*,*$Adv^{AKE}\left(M\right)$*is negligible in security parameter k.*

**Remark** **2.** *If a protocol is secure under Definition 3, then it achieves implicit mutual key authentication and the basic security properties, including weak perfect forward secrecy (wPFS), key compromise impersonation resilience (KCIR), ephemeral secrets reveal resistance (ESRR), known key security, no key control, resistance to basic impersonation attack, replay attack resilience, resistance to man-in-the-middle attack, and unknown key share resilience.*

## 4. Cryptanalysis of Several ID-AKA Protocols

**Figure 1:**
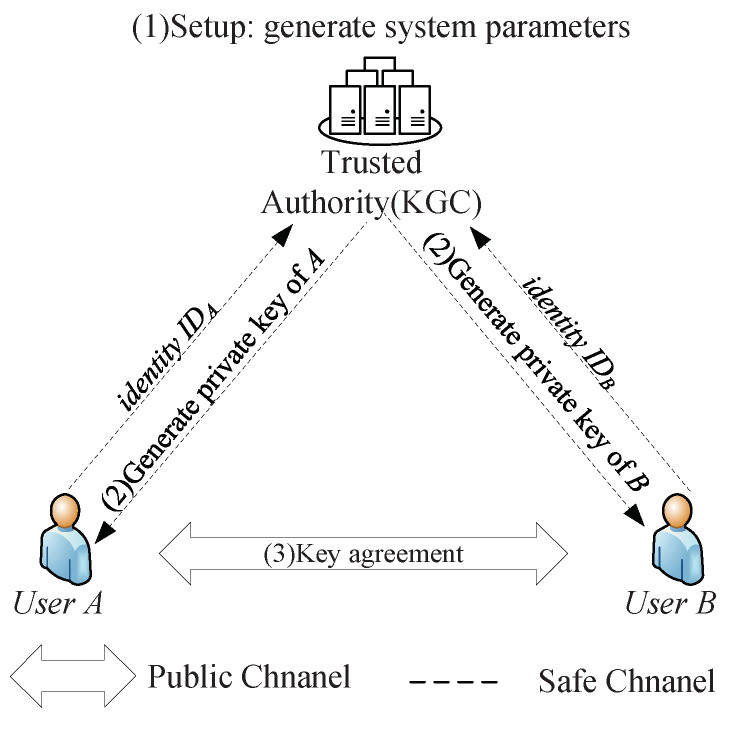
Network model for ID-AKA protocols.

Figure 1 shows the network model for ID-AKA protocols considered in our paper. Here, user *A*, user *B*, and trusted authority (acts as KGC) are the three main parties of an ID-AKA protocol. *A* and *B* obtain their long-term private keys in the extract phase and use them to reach AKA each other. Next, this section provides the cryptanalysis of several ID-AKA protocols.

### 4.1. The PF-ID-2PAKA Protocol

**Figure 2:**
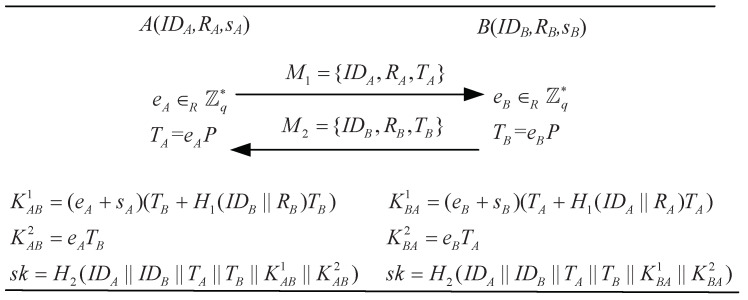
The key agreement phrase of the PF-ID-2PAKA protocol [21].

PF-ID-2PAKA [21] is also composed of three stages, i.e., setup, extract, and key agreement. The former two stages are the same as those of the DXCLCZF-18 protocol [28]. Here, we only describe the key agreement stage in Figure 2.

#### 4.1.1. Ephemeral Key Reveal Attack

Suppose that *A*’s ephemeral key $e_{A}$ is compromised by an adversary. Next, we show that the PF-ID-2PAKA protocol suffers from an ephemeral key reveal attack.

(1) The adversary M initializes a session $\prod_{A,B}^{\ell }$ through the query Send($\prod_{A,B}^{\ell },⊥$), and then obtains the message $M_{1}=\left\{ID_{A},R_{A},T_{A}\right\}$, where $T_{A}=e_{A}P$ with a random element $e_{A}\in Z_{q}^{*}$.
(2) Upon receiving the message, $M_{1}$, M randomly picks an ephemeral private key $e_{B}^{M}\in Z_{q}^{*}$, calculates $T_{B}^{M}=e_{B}^{M}P-R_{B}-H_{1}\left(ID_{B},R_{B}\right)P_{pub}$, and returns $M_{2}=\left\{ID_{B},R_{B},T_{B}^{M}\right\}$ to $\prod_{A,B}^{\ell }$ impersonating *B* via the query Send($\prod_{A,B}^{\ell },M_{2}$). Note that *B*’s identity $ID_{B}$ and correct public key material $R_{B}$ can be obtained from the response of the query Send($\prod_{B,U}^{t},⊥$).
(3) Upon the receipt of $M_{2}$, *A* calculates the shared session key and completes this session. Specifically, *A* calculates $sk=H_{2}(ID_{A}\left|\right|$$ID_{B}\left|\right|T_{A}\left|\right|T_{B}^{M}\left|\right|K_{AB}^{1}\left|\right|K_{AB}^{2})$, where $K_{AB}^{1}=\left(s_{A}+e_{A}\right)\left(R_{B}+H_{1}\left(ID_{B},R_{B}\right)P_{pub}+T_{B}^{M}\right)$ and $K_{AB}^{2}=e_{A}T_{B}^{M}.$
(4) Now, M performs the query EphemeralKeyReveal($\prod_{A,B}^{\ell }$) to reveal an ephemeral private key $e_{A}$. With the knowledge of $e_{B}^{M}$ and $e_{A}$, M computes $K_{BA}^{1M}=e_{B}^{M}\left(R_{A}+H_{1}\left(ID_{A},R_{A}\right)P_{pub}+T_{A}\right)$, $K_{BA}^{2M}=e_{A}T_{B}^{M}$, and $sk=H_{2}(ID_{A}\left|\right|$$ID_{B}\left|\right|T_{A}\left|\right|T_{B}^{M}\left|\right|K_{BA}^{1M}\left|\right|K_{BA}^{2M})$.

**Correctness.** The following provides the correctness of the attack process.

As a result of $T_{B}^{M}=e_{B}^{M}P-R_{B}-H_{1}\left(ID_{B},R_{B}\right)P_{pub}$, we can obtain

$$\begin{array}{cc} K_{AB}^{1} & =\left(s_{A}+e_{A}\right)\left(R_{B}+H_{1}\left(ID_{B},R_{B}\right)P_{pub}+T_{B}^{M}\right)\\ \; & =\left(s_{A}+e_{A}\right)e_{B}^{M}P\\ \; & =e_{B}^{M}\left(s_{A}P+e_{A}P\right)\\ \; & =e_{B}^{M}\left(R_{A}+H_{1}\left(ID_{A},R_{A}\right)P_{pub}+T_{A}\right)\\ \; & =K_{BA}^{1M}. \end{array}$$

Thus, *A* and M receive the same session key, which means that the PF-ID-2PAKA protocol suffers from an ephemeral key reveal attack. Note that our construction cannot suffer from the above attack as both of the two shared secrets not only depend on the ephemeral private key, but also depend on the long-term private key, and they are linearly independent. Therefore, it is impossible to remove the long-term private key from the two shared secrets simultaneously.

#### 4.1.2. Flaws in the Security Proof

The PF-ID-2PAKA protocol can not be proved secure under the hardness of the GDH problem in Case 3 (i.e., M can neither obtain the long-term private key of $ID_{A}$ nor that of $ID_{B}$). Meanwhile, the ephemeral key $t_{B}$ of $ID_{B}$ may be created by M, then CH cannot know $t_{B}$. In ignorance of $t_{B}$, CH does not calculate CDH$\left(U,V\right)=K_{1}-t_{A}\left(T_{B}+V\right)-t_{B}U$. Therefore, the challenger CH cannot solve the GDH instance.

### 4.2. The ZHWY-19 Protocol

**Figure 3:**
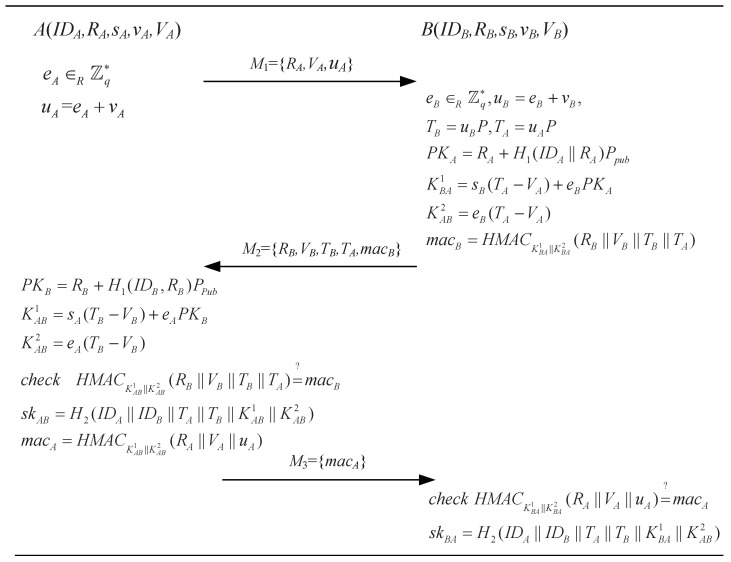
The key agreement phrase of the ZHWY-19-I protocol [23].

Here, we only describe the key agreement stage of the ZHWY-19-I protocol [23] in Figure 3. For more details, one can refer to [23]. Note that Cheng et al. [39] pointed out that the ZHWY-19-I protocol [23] cannot achieve forward security and resist the key compromise impersonation attack. Here, we point out that this protocol is weak against the ephemeral key reveal attack and flaws in the security proof.

#### 4.2.1. Ephemeral Key Reveal Attack

If $e_{A}$ and $e_{B}$ in a past session have been compromised by the adversary M, M can compute the session key sk.

(1) M accesses to $M_{1}=\left\{R_{A},V_{A},u_{A}\right\}$ and $M_{2}=\left\{R_{B},V_{B},T_{B},T_{A},mac_{B}\right\}$ of the session.
(2) Given {$ID_{A},e_{A},R_{A}$} and {$ID_{B},e_{B},R_{B}$}, M calculates the keys of $PK_{A}=R_{A}+H_{1}(ID_{A}\left|\right|$$R_{A})P_{pub},PK_{B}=R_{B}+H_{1}(ID_{B}\left|\right|R_{B})P_{pub}$, $K_{1}=e_{A}PK_{B}+e_{B}PK_{A}$, $K_{2}=e_{A}e_{B}P$, and $sk=H_{2}(ID_{A}||ID_{B}\left|\right|T_{A}\left|\right|T_{B}\left|\right|K_{1}\left|\right|K_{2})$ as the shared session key.

Thus, the ZHWY-19-I protocol is weak against the ephemeral secret key leakage. Note that they did not claim their protocol holds ephemeral key reveal resistance.

#### 4.2.2. Flaws in the Security Proof

In the ZHWY-19-I protocol, the answer to oracle Corrupt($ID_{i}$) is improper. Actually, Corrupt($ID_{i}$) should return the long-term private key $\left(s_{i},R_{i},v_{i},V_{i}\right)$ rather than $s_{i}$ to the adversary.

### 4.3. Mohammadali et al.’s Protocols

**Figure 4:**
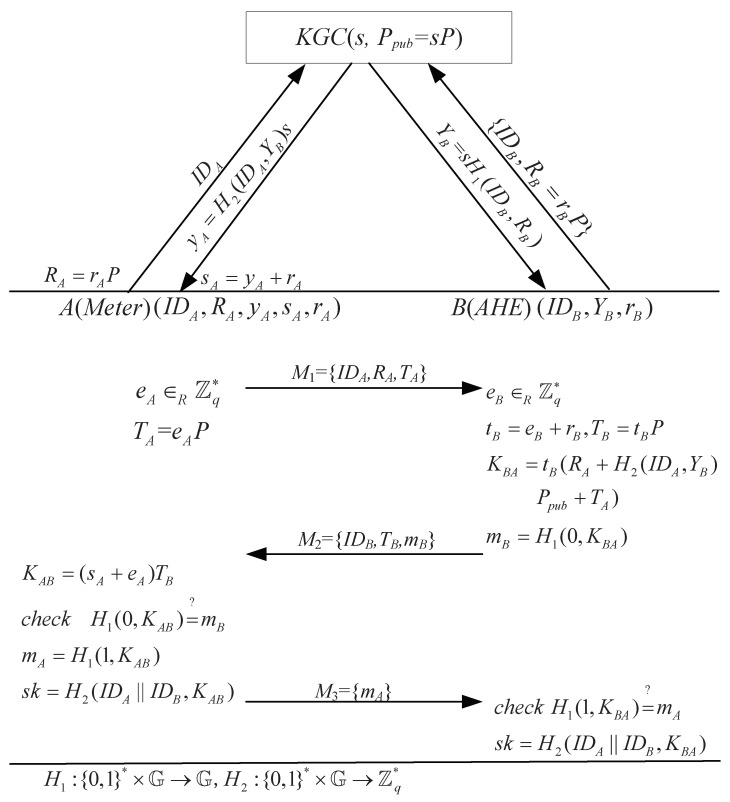
The NIKE protocol [22].

Mohammadali et al. [22] proposed two protocols, the NIKE protocol and the NIKE${}^{+}$ protocol. These two protocols contain three stages, namely setup, extract, and key agreement. The NIKE protocol is briefly shown in Figure 4. Note that, here, we did not analyze the flaws in the security proof as there was no security proof in [22].

#### 4.3.1. The Insecurity of the KGC’s Master Key

If the user A(Meter) and the user B(AHE) launch collusion attacks, they can know the KGC’s master key *s*. As $y_{A}=H_{2}\left(ID_{B},Y_{B}\right)s$, they can obtain $s=H_{2}{\left(ID_{B},Y_{B}\right)}^{-1}y_{A}$ with the knowledge of $y_{A}$ and $Y_{B}$.

#### 4.3.2. Key Compromise Impersonation (KCI) Attack

The NIKE protocol suffers from a key compromise impersonation (KCI) attack, i.e., if the user B(AHE)’s long-term private key $Y_{B}$ is compromised by M, M can impersonate any user (say A(Meter) ) with B(AHE)’s long-term private key $Y_{B}$ to communicate with B(AHE). The details are as follows. Note that they did not claim their protocol held KCI resistance.

(1) M obtains $ID_{A},R_{A}$ by eavesdropping on a connection between A(Meter) and any user. Then, M picks $e_{A}^{M}\in Z_{q}^{*}$ at random, calculates $T_{A}^{M}=e_{A}^{M}P-H_{2}\left(ID_{B},Y_{B}\right)P_{pub}-R_{A}$, and sends $\left\{ID_{A},T_{A}^{M},R_{A}\right\}$ to B(AHE).
(2) Upon the receipt of $\left\{ID_{A},T_{A}^{M},R_{A}\right\}$, B(AHE) generates $t_{B},T_{B},K_{BA},m_{B}$ according to the protocol.
(3) Upon receiving $\left\{ID_{B},T_{B},m_{B}\right\}$, M calculates $K_{AB}^{M}=e_{A}^{M}T_{B}$, $m_{A}^{M}=H_{1}\left(1,K_{AB}^{M}\right)$ and $SK=H_{1}(ID_{A}\left|\right|$$ID_{B}||,K_{AB}^{M})$, and finally sends $m_{A}^{M}$ to B(AHE).
(4) Upon receiving $m_{A}^{M}$, B(AHE) verifies $m_{A}^{M}$ is correct and computes the session key according to the protocol.

**Correctness**. The following provides the correctness of the attack process.

As $T_{A}^{M}=e_{A}^{M}P-H_{2}\left(ID_{B},y_{B}\right)P_{pub}-R_{A}$ and $T_{B}=t_{B}P$, we can obtain

$$\begin{array}{cc} K_{BA} & =t_{B}\left(R_{A}+H_{2}\left(ID_{A},Y_{B}\right)P_{pub}+T_{A}^{M}\right)\\ \; & =t_{B}(R_{A}+H_{2}\left(ID_{A},Y_{B}\right)P_{pub}+e_{A}^{M}P\\ \; & \;\;\;-H_{2}\left(ID_{B},Y_{B}\right)P_{pub}-R_{A})\\ \; & =t_{B}e_{A}^{M}P\\ \; & =e_{A}^{M}T_{B}\\ \; & =K_{AB}^{M}. \end{array}$$

Thus, *A* and M obtain the same session key, which means that the NIKE protocol suffers from a KCI attack. The KCI attack on the NIKE${}^{+}$ protocol is the same as above.

## 5. Our General Construction

This section firstly provides the construction $\sum_{C_{1},C_{2},C_{3},C_{4}}$, secondly show the construction $\sum_{C_{1},C_{2},C_{3},C_{4}}$ is correct, and thirdly provides the security proof.

### 5.1. Construction Description

The $\sum_{C_{1},C_{2},C_{3},C_{4}}$ is composed of three stages, i.e., setup, extract, and key agreement.

**Figure 5:**
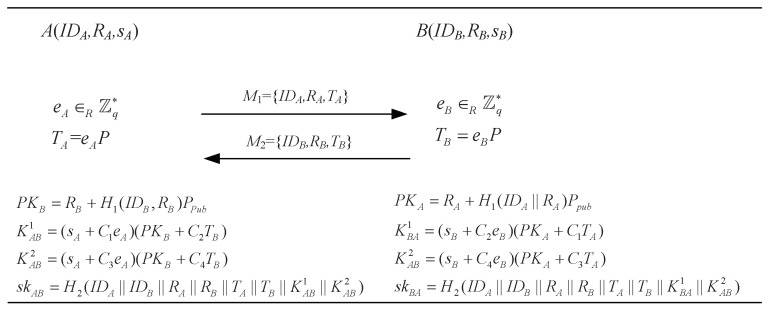
The key agreement phrase of our proposed construction $\sum_{C_{1},C_{2},C_{3},C_{4}}$.

- **Setup:** Select security parameter *k*, KGC performs as follows:
  (1) Pick an elliptic curve $E/F_{p}$, where $F_{p}$ is a finite filed, and *p* is a prime number with *k* bits.
  (2) Create a cyclic additive group G with the order *q*, which is generated by a base point *P* over $E/F_{p}$.
  (3) Choose $s\in Z_{q}^{*}$ randomly, and then set the master private key *s* and the system public key $P_{pub}=sP$.
  (4) Pick $H_{1}:{\left\{0,1\right\}}^{*}\to Z_{q}^{*}$ and $H_{2}:{\left\{0,1\right\}}^{*}\to {\left\{0,1\right\}}^{k}$.
  (5) Expose $\langle E/F_{p},G,q,P,P_{pub},H_{1},$$H_{2}\rangle$, and meanwhile retain *s* unrevealed.
- **Extract:** KGC derives the long-term private key for user $ID_{i}\in {\left\{0,1\right\}}^{*}$ as below.
  (1) KGC randomly selects $r_{i}\in Z_{q}^{*}$, and calculates $R_{i}=r_{i}P$ and $h_{i}=H_{1}(ID_{i}\left|\right|R_{i})$.
  (2) KGC computes $s_{i}=r_{i}+h_{i}s$ mod *q* and derives $\left(s_{i},R_{i}\right)$ as the user’s long-term private key.
  (3) KGC sends $\left(s_{i},R_{i}\right)$ to the user securely.
  Upon receiving $\left(s_{i},R_{i}\right)$, the user can verify $s_{i}P\overset{?}{=}R_{i}+H_{1}(ID_{i}\left|\right|R_{i})P_{pub}$. If this verification succeeds, the key pair $\left(s_{i},R_{i}\right)$ is correct and valid. $s_{i}P$ serves as the real public key in relation to $ID_{i}$.
- **Key Agreement:** Assume that user *A* with identity $ID_{A}$ hopes to compute a key with user *B* with identity $ID_{B}$.
  (1) *A* randomly picks an ephemeral secret key $e_{A}\in Z_{q}^{*}$, calculates $T_{A}=e_{A}P$, and returns $M_{1}=\left\{ID_{A},R_{A},T_{A}\right\}$ to *B*. The agreement process in Figure 5.
  (2) When $\left\{ID_{A},R_{A},T_{A}\right\}$ is received, *B* picks an ephemeral secret key $e_{B}\in Z_{q}^{*}$, calculates $T_{B}=e_{B}P$, and returns $\left\{ID_{B},R_{B},T_{B}\right\}$ to *A*. Next, *B* calculates $sk_{BA}=H_{2}(ID_{A}||ID_{B}\left|\right|R_{A}\left|\right|R_{B}\left|\right|T_{A}\left|\right|T_{B}\left|\right|K_{BA}^{1}\left|\right|K_{BA}^{2})$ as the shared session key, where $K_{BA}^{1}=\left(s_{B}+C_{2}e_{B}\right)\left(PK_{A}+C_{1}T_{A}\right)$, $K_{BA}^{2}=\left(s_{B}+C_{4}e_{B}\right)\left(PK_{A}+C_{3}T_{A}\right)$, $PK_{A}=R_{A}+H_{1}(ID_{A}\left|\right|R_{A})P_{pub}$, $C_{i}\left(i=1,2,3,4\right)\in Z_{q}^{*}$ and $C_{1}\neq C_{3},C_{2}\neq C_{4}$. Finally, *B* sends $M_{2}=\left\{ID_{B},R_{B},T_{B}\right\}$ to *A*.
  (3) When $M_{2}=\left\{ID_{B},R_{B},T_{B}\right\}$ is received, *A* calculates $PK_{B}=R_{B}+H_{1}(ID_{B}\left|\right|R_{B})P_{pub}$, and two shared secrets $K_{AB}^{1}=\left(s_{A}+C_{1}e_{A}\right)\left(PK_{B}+C_{2}T_{B}\right)$ and $K_{AB}^{2}=\left(s_{A}+C_{3}e_{A}\right)\left(PK_{B}+C_{4}T_{B}\right),$ where $C_{i}\left(i=1,2,3,4\right)\in Z_{q}^{*}$ and $C_{1}\neq C_{3},C_{2}\neq C_{4}$. Finally, *A* calculates the shared session key $sk_{AB}=H_{2}(ID_{A}||ID_{B}\left|\right|R_{A}\left|\right|R_{B}\left|\right|T_{A}\left|\right|T_{B}\left|\right|$$K_{AB}^{1}\left|\right|K_{AB}^{2})$.

Note that our construction provides a method to construct eCK secure ID-AKA protocols; however, parameters $C_{1},C_{2},C_{3}$, and $C_{4}$ should be fixed in the real execution environment. One can choose a concrete and efficient protocol derived from our construction to execute in the real environment, e.g., protocol $\sum_{1,1,-1,-1}$, protocol $\sum_{1,-1,-1,1}$ and protocol $\sum_{1,1,2,2}$ described in Section 6.

### 5.2. Construction Correctness

The following provides the correctness of our construction. As $PK_{B}=R_{B}+H_{1}(ID_{B}\left|\right|R_{B})$$P_{pub}=s_{B}P$, $PK_{A}=R_{A}+H_{1}(ID_{A}\left|\right|R_{A})P_{pub}=s_{A}P$, $T_{A}=e_{A}P$ and $T_{B}=e_{B}P$, we can obtain:

$$\begin{array}{cc} K_{AB}^{1} & =\left(s_{A}+C_{1}e_{A}\right)\left(PK_{B}+C_{2}T_{B}\right)\\ \; & =\left(s_{A}+C_{1}e_{A}\right)\left(s_{B}P+C_{2}e_{B}P\right)\\ \; & =\left(s_{A}+C_{1}e_{A}\right)\left(s_{B}+C_{2}e_{B}\right)P\\ \; & =\left(s_{B}+C_{2}e_{B}\right)\left(s_{A}+C_{1}e_{A}\right)P\\ \; & =\left(s_{B}+C_{2}e_{B}\right)\left(PK_{A}+C_{1}T_{A}\right)=K_{BA}^{1}=K_{1}; \end{array}$$

$$\begin{array}{cc} K_{AB}^{2} & =\left(s_{A}+C_{3}e_{A}\right)\left(PK_{B}+C_{4}T_{B}\right)\\ \; & =\left(s_{A}+C_{3}e_{A}\right)\left(s_{B}P+C_{4}e_{B}P\right)\\ \; & =\left(s_{A}+C_{3}e_{A}\right)\left(s_{B}+C_{4}e_{B}\right)P\\ \; & =\left(s_{B}+C_{4}e_{B}\right)\left(s_{A}+C_{3}e_{A}\right)P\\ \; & =\left(s_{B}+C_{4}e_{B}\right)\left(PK_{A}+C_{3}T_{A}\right)=K_{BA}^{2}=K_{2}. \end{array}$$

Thus both *A* and *B* compute $sk_{AB}=sk_{BA}=sk=H_{2}(ID_{A}||ID_{B}\left|\right|R_{A}\left|\right|R_{B}\left|\right|T_{A}\left|\right|T_{B}\left|\right|K_{1}\left|\right|$$K_{2})$ as their session key. Hence the correctness holds.

### 5.3. Security Proof

Here, the events in our security proof are complementary, while they are not complementary in [27], and the security proof can be reduced to the following theorems.

**Theorem** **1.** *Provide two random oracles, $H_{1}$ and $H_{2}$, the proposed ID-AKA protocol is secure in the eCK model based on the GDH assumption over the elliptic curve group.*

**Proof.**  This theorem is under the condition that the two conditions shown in Definition 3 hold. The correctness analysis shows that the first condition stands. The second condition would be proven by contradiction, i.e., there is an adversary who can execute a PPT algorithm to win the game with non-negligible probability, we can use M to create a GDH solver CH who can find a solution for the GDH instance. □

Assume M is a polynomially (in security parameter *k*) bounded adversary whose advantage is $Adv_{M}\left(k\right)$. Suppose that M activates no more than $n_{p}\left(k\right)$ different honest parties, and each party can take part in no more than $n_{s}\left(k\right)$ sessions. Suppose that M chooses $\prod_{a,b}^{n}$, the *n*th protocol session which executes between party $ID_{a}$ (the owner) and the target party $ID_{b}$ (the peer) as the test session. Assume that M performs, at most, $n_{h}\left(k\right)$$H_{2}$ queries.

According to $Adv^{AKE}\left(M\right)=\left|2Pr\left[M\;wins\right]-1\right|$, we can derive that Pr[Mwins] is non-negligible as $Adv^{AKE}\left(M\right)$ is non-negligible. As $H_{2}$ is modeled as a random oracle, M can make a clear distinction between a random string and the tested session key in the following three ways:

A1. Guessing attack: M directly guesses the correct session key.
A2. Key replication attack: M successfully creates a session that cannot match the test session while holding the same session key. Here, M can obtain the test session key by querying the non-matching session key.
A3. Forging attack: Sometimes, M makes $H_{2}$ queries on $(ID_{a},ID_{b},R_{a},R_{b},$$T_{a},T_{b},K_{1},K_{2})$ in the test session. Here, M calculates $K_{1}$ and $K_{2}$ itself.

The guessing of $H_{2}$’s output is with the negligible probability $O(1/2^{k})$. If two sessions are different, $H_{2}$ has the same input by probability $O(n_{s}{\left(k\right)}^{2}/2^{k})$, which is also negligible. Then, M can provide the difference between a random string and the tested session key only by *forging attack*.

Next, a reduction approach is applied to analyze the *forging attack*. This approach reduces the protocol security to the hardness of mathematical problems in the GDH assumption. By making assumptions about the adversary, a challenger can solve a GDH instance with the queried data and forged session key derived by a query-respond game between them. As the GDH instance cannot be solved with the current computation ability in polynomial time, the assumptions about the adversary are invalid, and the proposed ID-AKA protocols are secure.

Now, the detailed descriptions of the reduction proofs are as follows.

If M can successfully execute *forging attack* with non-negligible probability $Adv_{M}^{F}\left(k\right)$, we will use M to create a GDH solver CH to find a solution for the GDH instance with $Adv_{S}^{GDH}\left(k\right)$. Here, GDH instance is (U=uP,V=vP), and $u,v\in Z_{q}^{*},P\in G$, CH plans to calculate GDH(U,V)=uvP performing the DDH oracle. CH acts as a challenger that performs the eCK game with M and makes response for M’s queries.

Before the game starts, CH guesses the test session that M’s choices is $\prod_{a,b}^{n}$ with a correct probability at least $1/n_{p}{\left(k\right)}^{2}n_{s}\left(k\right)$. Next, CH needs to guess the strategy that M adopts. Then, according to Definition 2, test session $\prod_{a,b}^{n}$ has the matching session $\prod_{b,a}^{l}$, then M can only passively forward messages between participant $ID_{a}$ and participant $ID_{b}$, i.e., messages including public key materials and ephemeral keys of $\prod_{a,b}^{n}$ and $\prod_{b,a}^{l}$ are selected by CH. Test session $\prod_{a,b}^{n}$ has no matching session, then M alters some messages at its own will, i.e., messages including the public key material and the ephemeral key of $\prod_{a,b}^{n}$ are chosen by CH, and another one of $ID_{b}$ is chosen by M, and, thus, for $ID_{b}$, CH can only consider the long-term private key of $ID_{b}$. With the former analysis and the freeness definition, CH guesses the operation that M selects one of the following six complementary choices. Note that, strictly speaking, the ephemeral private key of $ID_{a}$ refers to $\prod_{a,b}^{n}$’s ephemeral private key, and the ephemeral private key of $ID_{b}$ refers to the matching session $\prod_{b,a}^{l}$’s ephemeral private key.

S1: $\prod_{a,b}^{n}$ has $\prod_{b,a}^{l}$, and M obtains neither the long-term private key of $ID_{a}$ nor the ephemeral private key of $ID_{b}$.
S2: $\prod_{a,b}^{n}$ has $\prod_{b,a}^{l}$, and M cannot obtain any information about the ephemeral private keys of $ID_{a}$ and $ID_{b}$.
S3: $\prod_{a,b}^{n}$ has $\prod_{b,a}^{l}$, and M knows neither the ephemeral private key of $ID_{a}$ nor the long-term private key of $ID_{b}$.
S4: $\prod_{a,b}^{n}$ has $\prod_{b,a}^{l}$, and M cannot obtain any information about the long-term private keys of $ID_{a}$ and $ID_{b}$.
S5: $\prod_{a,b}^{n}$ does not have a matching session, and M knows neither the ephemeral private key of $ID_{a}$ nor the long-term private key of $ID_{b}$.
S6: $\prod_{a,b}^{n}$ does not have a matching session, and M does not know any information about the long-term private keys of $ID_{a}$ and $ID_{b}$.

One of the former operation successes is if M succeeds in a forging attack with non-negligible probability. Therefore, the assumption about adversary M is invalid, and the proposed ID-AKA protocol is secure in the eCK model.

#### 5.3.1. The Analysis of Strategy S1

In this subsection, we analyze strategy S1.

- **Setup:** CH initializes a list ${Setup}^{list}$ with entries of $\left(ID_{i},\left(d_{i},R_{i}\right),PK_{i}\right)$. CH creates the system parameters and long-term private keys of all parties as follows.
  – CH picks $P_{pub}\in G$ at random, and exposes $\langle E/F_{p},G,q,P,P_{pub},H_{1},$$H_{2}\rangle$. Thus, CH cannot obtain any information about KGC’s master key.
  – For $ID_{a}$, CH sets the long-term private key $\left(⊥,R_{a}\right)$, where $h_{a}\in_{R}Z_{q}^{*}$, $R_{a}=U-h_{a}P_{pub}$. Thus, $PK_{a}=R_{a}+h_{a}P_{pub}=U$.
  – For $ID_{i}\left(i\neq a\right)$, CH sets the long-term private key $\left(s_{i},R_{i}\right)$, where $h_{i},s_{i}\in_{R}Z_{q}^{*}$, $R_{i}=s_{i}P-h_{i}P_{pub}$. Thus, $PK_{i}=R_{i}+h_{i}P_{pub}=s_{i}P$.
  – For every participant, CH transfers $\left(ID_{i},R_{i}\right)$ to M, and stores the tuple $(ID_{i},\left(d_{i},R_{i}\right),$$PK_{i})$ and $\left(ID_{i},R_{i},h_{i}\right)$ in ${Setup}^{list}$ and ${H_{1}}^{list}$ (described later), respectively.
- **Queries:** CH maintains four lists, ${H_{1}}^{list}$, ${H_{2}}^{list}$, ${Send}^{list}$, and $R^{list}$, which are initially empty and used to record $H_{1}$, $H_{2}$, Send, and SessionKeyReveal oracles, respectively. CH starts M by answering M’s queries, as follows.
  – $H_{1}\left(ID_{i},R_{i}\right)$: If an entry $\left(ID_{i},R_{i},h_{i}\right)$ is recorded in ${H_{1}}^{list}$, CH responds with $h_{i}$. Then, CH randomly selects $h_{i}\in Z_{q}^{*}$, appends $\left(ID_{i},R_{i},h_{i}\right)$ to ${H_{1}}^{list}$, and sends $h_{i}$ back to M.
  – $H_{2}\left(ID_{i},ID_{j},R_{i},R_{j},T_{i},T_{j},K_{1},K_{2}\right)$: List ${H_{2}}^{list}$ is with $(ID_{i},ID_{j},$$R_{i},R_{j},T_{i},T_{j},K_{1},K_{2},h_{2})$.
    * If a matching entry $(ID_{i},ID_{j},R_{i},R_{j},$$T_{i},T_{j},K_{1},K_{2},h_{2})$ is stored in ${H_{2}}^{list}$, CH replies with $h_{2}$.
    * Else, CH seeks $(*,ID_{i},ID_{j},$$R_{i},R_{j},T_{i},T_{j},*)$ in $R^{list}$. Then, if such an entry exists, CH sees if $K_{1}$ and $K_{2}$ are produced correctly by validating DDH($PK_{i}+C_{1}T_{i},PK_{j}+C_{2}T_{j},K_{1})\overset{?}{=}1$ and DDH($PK_{i}+C_{3}T_{i},PK_{j}+C_{4}T_{j},K_{2})\overset{?}{=}1$, respectively, where $PK_{i}=R_{i}+H_{1}\left(ID_{i},R_{i}\right)P_{pub}$ and $PK_{j}=R_{j}+H_{1}\left(ID_{j},R_{j}\right)P_{pub}$. If both verifications pass, CH receives the corresponding $SK_{i,j}^{m}$ and sets $h_{2}\leftarrow SK_{i,j}^{m}$. Otherwise (at least one verification fails or none), CH picks $h_{2}\in {\left\{0,1\right\}}^{k}$ at random. Finally, CH inserts the tuple $\left(ID_{i},ID_{j},T_{i},T_{j},K_{1},K_{2},h_{2}\right)$ into ${H_{2}}^{list}$ and provides $h_{2}$ as the answer.
  – Corrupt($ID_{i}$): If $ID_{i}=ID_{a}$, CH discontinues. Otherwise, CH responds with $s_{i}$.
  – KGCStaticKeyReveal: CH discontinues.
  – EphemeralKeyReveal($\prod_{i,j}^{m}$): If $\prod_{i,j}^{m}=\prod_{b,a}^{l}$, CH discontinues. Otherwise, CH provides the stored ephemeral key $r_{i,j}^{m}$ as the answer.
  – Send($\prod_{i,j}^{m}$,*M*): List ${Send}^{list}$ is with $\left(\prod_{i,j}^{m},tran_{i,j}^{m},r_{i,j}^{m},State_{i,j}^{m}\right)$, where $tran_{i,j}^{m}$, $r_{i,j}^{m}$ and $State_{i,j}^{m}$ are the transcript by now, the ephemeral secret key, and the state by now, respectively.
    * If *M* is the second message on the transcript, CH sets $State_{i,j}^{m}=completed$ and updates ${Send}^{list}$.
    * Else CH executes as follows.
      · If $\prod_{i,j}^{m}=\prod_{b,a}^{l}$, CH sets $r_{i,j}^{m}=⊥$, gets $R_{b}$ from ${Setup}^{list}$ and replies with $\left\{ID_{b},R_{b},V\right\}$.
      · Else CH randomly chooses $r_{i,j}^{m}\in Z_{q}^{*}$, obtains $R_{i}$ from ${Setup}^{list}$ and replies with $\left\{ID_{i},R_{i},r_{i,j}^{m}P\right\}$.
      · Finally, CH updates ${Send}^{list}$, and updates $State_{i,j}^{m}$ to completed if the newly generated message is the second message on the transcript.
  – SessionKeyReveal($\prod_{i,j}^{m}$): List $R^{list}$ is of the form $(\prod_{i,j}^{m},ID_{ini},ID_{resp},R_{ini},R_{resp},T_{ini},$$T_{resp},SK_{i,j}^{m})$, where ini∈{i,j} and resp∈{i,j} denote the initiator and the responder of $\prod_{i,j}^{m}$, respectively.
    * CH receives $State_{i,j}^{m}$ from ${Send}^{list}$. If $State_{i,j}^{m}\neq completed$, CH returns *⊥*.
    * Else if $\prod_{i,j}^{m}=\prod_{a,b}^{n}$ or $\prod_{i,j}^{m}=\prod_{b,a}^{l}$, CH aborts.
    * Else if the session key $SK_{i,j}^{m}$ already exists, CH responds with $SK_{i,j}^{m}$.
    * Else CH obtains $\left\{ID_{ini},R_{ini},T_{ini}\right\}$ and $\left\{ID_{resp},R_{resp},T_{resp}\right\}$ from ${Send}^{list}$, and looks up ${H_{2}}^{list}$ to see if there is a tuple $(*,ID_{ini},ID_{resp},$$R_{ini},R_{resp},T_{ini},T_{resp},*)$. Then, if it exists, CH sees if $K_{1}$ and $K_{2}$ are produced correctly by validating DDH($PK_{ini}+C_{1}T_{ini},PK_{resp}+C_{3}T_{resp},K_{1})\overset{?}{=}1$ and DDH($PK_{ini}+C_{3}T_{ini},PK_{resp}$$+C_{4}T_{resp},K_{2})\overset{?}{=}1$, respectively, where $PK_{ini}=R_{ini}+H_{1}\left(ID_{ini},R_{ini}\right)P_{pub}$ and $PK_{resp}=R_{resp}+H_{1}\left(ID_{resp},R_{resp}\right)P_{pub}$. If both verifications pass, CH receives the corresponding $h_{2}$ and sets $SK_{i,j}^{m}\leftarrow h_{2}$. Otherwise (at least one verification fails or no such a tuple exists), CH picks $SK_{i,j}^{m}\in {\left\{0,1\right\}}^{k}$ at random. Finally, CH inserts the tuple $(\prod_{i,j}^{m},ID_{ini},ID_{resp},R_{ini},R_{resp},T_{ini},T_{resp},$$SK_{i,j}^{m})$ into $R^{list}$ and returns $SK_{i,j}^{m}$.
  – Test($\prod_{i,j}^{m}$): If $\prod_{i,j}^{m}=\prod_{a,b}^{n}$, CH picks $\xi \in {\left\{0,1\right\}}^{k}$ at random and sends ξ back to M. Otherwise, CH aborts.
- **Analysis:** If M can successfully execute a forging attack in Strategy S1 with non-negligible probability, the following conditions should be met.
  (1) CH continues following the above simulation. If M chooses Strategy S1, with $\prod_{a,b}^{n}$ and $\prod_{b,a}^{l}$ as the test session and its corresponding matching session, respectively, this condition can be met.
  (2) For the test session $\prod_{a,b}^{n}$, adversary M must have conducted $H_{2}$ queries on the values $\{ID_{a},ID_{b},R_{a},R_{b},T_{a},$$V,K_{1},K_{2}\}$, where $R_{a}$ and $R_{b}$ are the public key materials of $ID_{a}$ and $ID_{b}$ picked by the challenger CH, respectively, $T_{a}$ and *V* are the outgoing messages of $ID_{a}$ and $ID_{b}$ picked by the challenger CH, respectively, and $K_{1}$ and $K_{2}$ are correctly formed.
    * If $\prod_{a,b}^{n}$ is an initiator, the correct input of $H_{2}$ should be $(ID_{a},ID_{b},R_{a},R_{b},T_{a},V,$$K_{1},K_{2})$, where $K_{1}=\left(DLOG\left(U\right)+C_{1}r_{a,b}^{n}\right)\left(R_{b}+h_{b}P_{pub}+C_{2}V\right)$, $K_{2}=(DLOG$$\left(U\right)+C_{3}r_{a,b}^{n})\left(R_{b}+h_{b}P_{pub}+C_{4}V\right)$, and $h_{b}=H_{1}\left(ID_{b},R_{b}\right)$.
    * If $\prod_{a,b}^{n}$ is a responder, the correct input of $H_{2}$ should be $(ID_{b},ID_{a},R_{b},R_{a},V,T_{a},$$K_{1},K_{2})$, where $K_{1}=\left(DLOG\left(U\right)+C_{2}r_{a,b}^{n}\right)\left(R_{b}+h_{b}P_{pub}+C_{1}V\right)$, $K_{2}=(DLOG$$\left(U\right)+C_{4}r_{a,b}^{n})\left(R_{b}+h_{b}P_{pub}+C_{3}V\right)$, and $h_{b}=H_{1}\left(ID_{b},R_{b}\right)$.
  Finally, CH receives the item in ${H_{2}}^{list}$ and outputs GDH$\left(U,V\right)={\left(C_{2}-C_{4}\right)}^{-1}(K_{1}-K_{2}+r_{a,b}^{n}\left(C_{3}-C_{1}\right)\left(R_{b}+h_{b}P_{pub}\right)$$+r_{a,b}^{n}\left(C_{3}C_{4}-C_{1}C_{2}\right)V)$ if $\prod_{a,b}^{n}$ is an initiator or GDH$\left(U,V\right)={\left(C_{1}-C_{3}\right)}^{-1}(K_{1}-K_{2}+r_{a,b}^{n}\left(C_{4}-C_{2}\right)\left(R_{b}+h_{b}P_{pub}\right)$$+r_{a,b}^{n}\left(C_{3}C_{4}-C_{1}C_{2}\right)V)$ if $\prod_{a,b}^{n}$ is a responder by the knowledge of $r_{a,b}^{n}$. Note that since $C_{i}\left(i=1,2,3,4\right)\in Z_{q}^{*}$ and $C_{1}\neq C_{3},C_{2}\neq C_{4}$, the solution of GDH(U,V) is correct. The CH success rate is at least
  $$Adv_{S}^{GDH}\left(k\right)>=\frac{Adv_{M}^{F}\left(k\right)}{6n_{h}\left(k\right)n_{p}{\left(k\right)}^{2}n_{s}{\left(k\right)}^{2}}.$$
  As $Adv_{M}^{F}\left(k\right)$ is non-negligible, $Adv_{S}^{GDH}\left(k\right)$ can also be seen as non-negligible. Now, it derives the contradiction of the GDH assumption.

#### 5.3.2. The Analysis of Strategy S2

In this subsection, we analyze strategy S2.

- **Setup:** ${Setup}^{list}$ is an initially empty list with $(ID_{i},\left(d_{i},R_{i}\right),$$PK_{i})$. CH creates the system parameters and all parties’ long-term private keys as follows.
  – CH picks $s\in Z_{q}^{*}$ at random, computes $P_{pub}=sP$, and exposes system parameters $\langle E/F_{p},G,q,P,P_{pub},$$H_{1},H_{2}\rangle$. Thus, CH cannot obtain any information about KGC’s master key.
  – For $ID_{i}$, CH sets the long-term private key $\left(s_{i},R_{i}\right)$, where $h_{i},s_{i}\in_{R}Z_{q}^{*}$, $R_{i}=s_{i}P-h_{i}P_{pub}$. Thus, $PK_{i}=R_{i}+h_{i}P_{pub}=s_{i}P$.
  – For every participant, CH transfers $\left(ID_{i},R_{i}\right)$ to M, and stores the tuple $(ID_{i},\left(d_{i},R_{i}\right),$$PK_{i})$ and $\left(ID_{i},R_{i},h_{i}\right)$ in ${Setup}^{list}$ and ${H_{1}}^{list}$ (described later), respectively.
- **Queries:** CH maintains four lists ${H_{1}}^{list}$, ${H_{2}}^{list}$, ${Send}^{list}$, and $R^{list}$ to store $H_{1}$, $H_{2}$, Send, and SessionKeyReveal oracles, respectively. CH performs the queries game with M as follows:
  – $H_{1}\left(ID_{i},R_{i}\right)$, SessionKeyReveal($\prod_{i,j}^{m}$), Test($\prod_{i,j}^{m}$), and $H_{2}(ID_{i},ID_{j},R_{i},R_{j},T_{i},T_{j},K_{1},$$K_{2})$: These four queries are described in the same as those in Strategy S1.
  – Corrupt($ID_{i}$): CH responds with $\left(s_{i},R_{i}\right)$.
  – KGCStaticKeyReveal: CH responds with *s* to M.
  – EphemeralKeyReveal($\prod_{i,j}^{m}$): If $\prod_{i,j}^{m}=\prod_{a,b}^{n}$ or $\prod_{i,j}^{m}=\prod_{b,a}^{l}$, CH discontinues. Otherwise, CH provides the stored ephemeral key $r_{i,j}^{m}$ as the answer.
  – Send($\prod_{i,j}^{m}$,*M*): List ${Send}^{list}$ has $\left(\prod_{i,j}^{m},tran_{i,j}^{m},r_{i,j}^{m},State_{i,j}^{m}\right)$, where $tran_{i,j}^{m}$, $r_{i,j}^{m}$ and $State_{i,j}^{m}$ are the transcript by now, the ephemeral secret key, and the state by now, respectively.
    * If *M* is the second message on the transcript, CH sets $State_{i,j}^{m}=completed$ and updates ${Send}^{list}$.
    * Else CH performs the following steps.
      · If $\prod_{i,j}^{m}=\prod_{a,b}^{n}$, CH sets $r_{i,j}^{m}=⊥$, receives $R_{a}$ from ${Setup}^{list}$, and replies with $\left\{ID_{a},R_{a},U\right\}$.
      · Else If $\prod_{i,j}^{m}=\prod_{b,a}^{l}$, CH sets $r_{i,j}^{m}=⊥$, receives $R_{b}$ from ${Setup}^{list}$, and replies with $\left\{ID_{b},R_{b},V\right\}$.
      · Else CH randomly chooses $r_{i,j}^{m}\in Z_{q}^{*}$, obtains $R_{i}$ from ${Setup}^{list}$, and replies with $\left\{ID_{i},R_{i},r_{i,j}^{m}P\right\}$.
      · Finally, CH updates ${Send}^{list}$, and updates $State_{i,j}^{m}$ to completed if the newly generated message is the second message on the transcript.
- **Analysis:** Here, we assume that $\prod_{a,b}^{n}$ is an initiator here. If M indeed chooses Strategy S2, $\prod_{a,b}^{n}$ and $\prod_{b,a}^{l}$ as the test session and its matching session, respectively, then CH continues this simulation. If M successfully executes the forging attack, it must have queried oracle $H_{2}(ID_{a},ID_{b},R_{a},R_{b},U,$$V,K_{1},K_{2})$, where $R_{a},R_{b},U$, and *V* are all picked by the challenger CH, $K_{1}=\left(s_{a}+C_{1}DLOG\left(U\right)\right)\left(R_{b}+h_{b}P_{pub}+C_{2}V\right)$, $K_{2}=\left(s_{a}+C_{3}DLOG\left(U\right)\right)\left(R_{b}+h_{b}P_{pub}+C_{4}V\right)$, and $h_{b}=H_{1}\left(ID_{b},R_{b}\right)$.
  Finally, CH receives the item in ${H_{2}}^{list}$, and outputs GDH$\left(U,V\right)={\left(C_{1}C_{3}\left(C_{2}-C_{4}\right)\right)}^{-1}(C_{3}K_{1}$$-C_{1}K_{2}+s_{a}\left(C_{1}-C_{3}\right)\left(R_{b}+h_{b}P_{pub}\right)+s_{a}\left(C_{1}C_{4}-C_{2}C_{3}\right)V)$ by the knowledge of $s_{a}$. Note that as $C_{i}\left(i=1,2,3,4\right)\in Z_{q}^{*}$ and $C_{1}\neq C_{3},C_{2}\neq C_{4}$, the solution of GDH(U,V) is correct. CH’s success probability is at least
  $$Adv_{S}^{GDH}\left(k\right)>=\frac{Adv_{M}^{F}\left(k\right)}{6n_{h}\left(k\right)n_{p}{\left(k\right)}^{2}n_{s}{\left(k\right)}^{2}}.$$
  As $Adv_{M}^{F}\left(k\right)$ is non-negligible, $Adv_{S}^{GDH}\left(k\right)$ can also be seen as also non-negligible. Now, it derives the contradiction of the GDH assumption.

#### 5.3.3. The Analysis of Strategy S3

Here, we omit the detailed analysis of Strategy S3 as the analysis is almost the same as that for Strategy S1.

#### 5.3.4. The Analysis of Strategy S4

In this subsection, we analyze strategy S4.

- **Setup:** CH initializes a list ${Setup}^{list}$ with $(ID_{i},\left(d_{i},R_{i}\right),$$PK_{i})$. CH creates the system parameters and all parties’ long-term private keys.
  – CH picks $P_{pub}\in G$ at random, and exposes $\langle E/F_{p},G,$$q,P,P_{pub},H_{1},$$H_{2}\rangle$. Thus, CH does not obtain any information about KGC’s master key.
  – For $ID_{a}$, CH sets the long-term private key $\left(⊥,R_{a}\right)$, where $h_{a}\in_{R}Z_{q}^{*}$, $R_{a}=U-h_{a}P_{pub}$. Thus, $PK_{a}=R_{a}+h_{a}P_{pub}=U$.
  – For $ID_{b}$, CH sets the long-term private key $\left(⊥,R_{b}\right)$, where $h_{b}\in_{R}Z_{q}^{*}$, $R_{b}=V-h_{b}P_{pub}$. Thus, $PK_{b}=R_{b}+h_{b}P_{pub}=V$.
  – For $ID_{i}\left(i\neq a,i\neq b\right)$, CH sets the long-term private key $\left(s_{i},R_{i}\right)$, where $h_{i},s_{i}\in_{R}Z_{q}^{*}$, $R_{i}=s_{i}P-h_{i}P_{pub}$. Thus, $PK_{i}=R_{i}+h_{i}P_{pub}=s_{i}P$.
  – For every participant, CH transfers $\left(ID_{i},R_{i}\right)$ to M, and stores $\left(ID_{i},\left(d_{i},R_{i}\right),PK_{i}\right)$ and $\left(ID_{i},R_{i},h_{i}\right)$ in ${Setup}^{list}$ and ${H_{1}}^{list}$ (described later), respectively.
- **Queries:** CH maintains four lists ${H_{1}}^{list}$, ${H_{2}}^{list}$, ${Send}^{list}$, and $R^{list}$, which are initially empty and used for recording $H_{1}$, $H_{2}$, Send, and SessionKeyReveal oracles, respectively. CH performs the queries game with M as follows:
  – $H_{1}\left(ID_{i},R_{i}\right)$, SessionKeyReveal($\prod_{i,j}^{m}$), Test($\prod_{i,j}^{m}$), $H_{2}(ID_{i},ID_{j},R_{i},R_{j},$$T_{i},T_{j},K_{1},K_{2})$, and KGCStaticKeyReveal: These five queries are described in the same way as those in Strategy S1.
  – Corrupt($ID_{i}$): If $ID_{i}=ID_{a}$ or $ID_{i}=ID_{b}$, CH discontinues. Otherwise, CH responds with $\left(s_{i},R_{i}\right)$.
  – EphemeralKeyReveal($\prod_{i,j}^{m}$): CH responses with $r_{i,j}^{m}$.
  – Send($\prod_{i,j}^{m}$,*M*): List ${Send}^{list}$ is of the form $\left(\prod_{i,j}^{m},tran_{i,j}^{m},r_{i,j}^{m},State_{i,j}^{m}\right)$, where $tran_{i,j}^{m}$, $r_{i,j}^{m}$, and $State_{i,j}^{m}$ are the transcript by now, the ephemeral secret key, and the state by now, respectively.
    * If *M* is the second message on the transcript, CH sets $State_{i,j}^{m}=completed$ and updates ${Send}^{list}$.
    * Else CH randomly chooses $r_{i,j}^{m}\in Z_{q}^{*}$, obtains $R_{i}$ from ${Setup}^{list}$, and replies with $\left\{ID_{i},R_{i},r_{i,j}^{m}P\right\}$. Then, CH updates ${Send}^{list}$, and updates $State_{i,j}^{m}$ to completed if the newly generated message is the second message on the transcript.
- **Analysis:** Here, we assume that $\prod_{a,b}^{n}$ is an initiator. If M selects Strategy S4, $\prod_{a,b}^{n}$ and $\prod_{b,a}^{l}$ as the test session and its matching session, then CH does not abort in the simulation. If M successfully performs the forging attack, it must have queried oracle $H_{2}\left(ID_{a},ID_{b},U-h_{a}P_{pub},V-h_{b}P_{pub},T_{a},T_{b},K_{1},K_{2}\right)$, where $T_{a},T_{b},U$, and *V* are all picked by the challenger CH, $K_{1}=\left(DLOG\left(U\right)+C_{1}r_{a,b}^{n}\right)\left(V+C_{2}T_{b}\right)$, $K_{2}=\left(DLOG\left(U\right)+C_{3}r_{a,b}^{n}\right)\left(V+C_{4}T_{b}\right)$, $h_{a}=H_{1}\left(ID_{a},R_{a}\right)$, and $h_{b}=H_{1}\left(ID_{b},R_{b}\right)$.
  Finally, CH receives the item in ${H_{2}}^{list}$, and outputs GDH$\left(U,V\right)={\left(C_{4}-C_{2}\right)}^{-1}(C_{4}K_{1}-C_{2}K_{2}+r_{a,b}^{n}\left(C_{2}C_{3}-C_{1}C4\right)V$$+r_{a,b}^{n}C_{2}C_{4}\left(C_{3}-C_{1}\right)T_{b})$ by the knowledge of $r_{a,b}^{n}$. Note that as $C_{i}\left(i=1,2,3,4\right)\in Z_{q}^{*}$ and $C_{1}\neq C_{3},C_{2}\neq C_{4}$, the solution of GDH(U,V) is correct. CH’s success probability is at least
  $$Adv_{S}^{GDH}\left(k\right)>=\frac{Adv_{M}^{F}\left(k\right)}{6n_{h}\left(k\right)n_{p}{\left(k\right)}^{2}n_{s}{\left(k\right)}^{2}}.$$
  As $Adv_{M}^{F}\left(k\right)$ is non-negligible, $Adv_{S}^{GDH}\left(k\right)$ can also be seen as non-negligible. Now, it derives the contradiction of the GDH assumption.

#### 5.3.5. The Analysis of Strategy S5

$\prod_{a,b}^{n}$ has no matching session in strategy S5, thus at least one of $ID_{b}$’s public key material $R_{b}$ and $ID_{b}$’s ephemeral private key is chosen by M. If the adversary selects $R_{b}$ themselves, then the change in $R_{b}$ means the change in the $ID_{b}$’s long-term private key $s_{b}$. Hence, a GDH instance cannot be embedded in the long-term private key in strategy S5.

- **Setup:** ${Setup}^{list}$ is an initially empty list and $(ID_{i},\left(d_{i},R_{i}\right),$$PK_{i})$ is needed in this phase. CH creates the system parameters and all parties’ long-term private keys.
  – CH sets *V* as the system public key $P_{pub}$ and exposes the system parameters $\langle E/F_{p},G,$$q,P,P_{pub},H_{1},H_{2}\rangle$. Thus CH cannot know KGC’s master key.
  – For $ID_{i}$, CH sets the long-term private key $\left(s_{i},R_{i}\right)$, where $h_{i},s_{i}\in_{R}Z_{q}^{*}$, $R_{i}=s_{i}P-h_{i}P_{pub}$. Thus, $PK_{i}=R_{i}+h_{i}P_{pub}=s_{i}P$.
  – For every participant, CH transfers $\left(ID_{i},R_{i}\right)$ to M, and stores $(ID_{i},$$\left(d_{i},R_{i}\right),PK_{i})$ and $\left(ID_{i},R_{i},h_{i}\right)$ in ${Setup}^{list}$ and ${H_{1}}^{list}$ (described later), respectively.
- **Queries:** CH maintains four lists, ${H_{1}}^{list}$, ${H_{2}}^{list}$, ${Send}^{list}$, and $R^{list}$ to store $H_{1}$, $H_{2}$, Send, and SessionKeyReveal oracles, respectively. CH performs the queries game with M as follows:
  – $H_{1}\left(ID_{i},R_{i}\right)$, KGCStaticKeyReveal, Test($\prod_{i,j}^{m}$), and $H_{2}(ID_{i},ID_{j},R_{i},$$R_{j},T_{i},T_{j},K_{1},K_{2})$ are described the same as those in Strategy S1.
  – Corrupt($ID_{i}$): If $ID_{i}=ID_{b}$, CH discontinues. Otherwise, CH responds with $\left(s_{i},R_{i}\right)$.
  – EphemeralKeyReveal($\prod_{i,j}^{m}$): If $\prod_{i,j}^{m}=\prod_{a,b}^{n}$, CH discontinues. Otherwise, CH provides the stored ephemeral key $r_{i,j}^{m}$ as the answer.
  – Send($\prod_{i,j}^{m}$,*M*): List ${Send}^{list}$ is with $\left(\prod_{i,j}^{m},tran_{i,j}^{m},r_{i,j}^{m},State_{i,j}^{m}\right)$, where $tran_{i,j}^{m}$, $r_{i,j}^{m}$, and $State_{i,j}^{m}$ are the transcript by now, the ephemeral secret key, and the state by now, respectively.
    * If *M* is the second message on the transcript, CH sets $State_{i,j}^{m}=completed$ and updates ${Send}^{list}$.
    * Else CH performs the following steps.
      · If $\prod_{i,j}^{m}=\prod_{a,b}^{n}$, CH sets $r_{i,j}^{m}=⊥$, receives $R_{a}$ from ${Setup}^{list}$, and replies with $\left\{ID_{a},R_{a},U\right\}$.
      · Else CH randomly chooses $r_{i,j}^{m}\in Z_{q}^{*}$, obtains $R_{i}$ from ${Setup}^{list}$, and replies with $\left\{ID_{i},R_{i},r_{i,j}^{m}P\right\}$.
      · Finally, CH updates ${Send}^{list}$, and updates $State_{i,j}^{m}$ to completed if the newly generated message is the second message on the transcript.
  – SessionKeyReveal($\prod_{i,j}^{m}$): This query is the same as that in Strategy S1, except that “Else if $\prod_{i,j}^{m}=\prod_{a,b}^{n}$ or $\prod_{i,j}^{m}=\prod_{b,a}^{l}$” is modified to “Else if $\prod_{i,j}^{m}=\prod_{a,b}^{n}$”. This is because the matching session $\prod_{b,a}^{l}$ certainly exists in Strategy S1, while $\prod_{b,a}^{l}$ does not exist in Strategy S5.
- **Analysis:** Here, we assume that $\prod_{a,b}^{n}$ is an initiator. If M selects Strategy S5 and $\prod_{a,b}^{n}$ as the test session, then CH continues using the above simulation. If M successfully performs the forging attack with non-negligible probability, it should execute the $H_{2}$ query on $\left(ID_{a},ID_{b},R_{a},R_{b},U,T_{b},K_{1},K_{2}\right)$, where $K_{1}=\left(s_{a}+C_{1}DLOG\left(U\right)\right)\left(R_{b}+h_{b}V+C_{2}T_{b}\right)$, $K_{2}=\left(s_{a}+C_{3}DLOG\left(U\right)\right)\left(R_{b}+h_{b}V+C_{4}T_{b}\right)$, and $h_{b}=H_{1}\left(ID_{b},R_{b}\right)$. Note that $ID_{a}$’s public key material $R_{a}$ and outgoing message *U* are both picked by the challenger CH, and at least one of $ID_{b}$’s public key material $R_{b}$ and outgoing message $T_{b}$ is chosen by M.
  By the forking lemma [27], CH replays M with the same input and tossing coins. Here, CH only changes the query results of $H_{1}\left(ID_{b},R_{b}\right)$, i.e., CH sets $H_{1}\left(ID_{b},R_{b}\right)$ to $\bar{h_{b}}$, where $\bar{h_{b}}\in Z_{q}^{*}$ and $\bar{h_{b}}\neq h_{b}$. Then, if M succeeds, it should perform a query on $H_{2}$ with $\left(ID_{a},ID_{b},R_{a},R_{b},U,T_{b},\bar{K_{1}},\bar{K_{2}}\right)$, where $\bar{K_{1}}=\left(s_{a}+C_{1}DLOG\left(U\right)\right)\left(R_{b}+\bar{h_{b}}V+C_{2}T_{b}\right)$, $\bar{K_{2}}=\left(s_{a}+C_{3}DLOG\left(U\right)\right)$$\left(R_{b}+\bar{h_{b}}V+C_{4}T_{b}\right)$.
  Finally, CH receives the item in ${H_{2}}^{list}$, and outputs GDH$\left(U,V\right)=C_{1}^{-1}\left({\left(h_{b}-\bar{h_{b}}\right)}^{-1}\left(K_{1}-\bar{K_{1}}\right)-s_{a}V\right)$ using the knowledge of $s_{a}$. Note that as $C_{1}\in Z_{q}^{*}$, the solution of GDH(U,V) is correct. Let λ be a factor from the forking lemma for Strategy S5. CH’s success probability is at least
  $$Adv_{S}^{GDH}\left(k\right)>=\frac{\lambda Adv_{M}^{F}\left(k\right)}{6n_{h}{\left(k\right)}^{2}n_{p}{\left(k\right)}^{2}n_{s}\left(k\right)}.$$
  As $Adv_{M}^{F}\left(k\right)$ is non-negligible, $Adv_{S}^{GDH}\left(k\right)$ can also be seen as non-negligible. Now, it derives the contradiction of the GDH assumption.

#### 5.3.6. The Analysis of Strategy S6

In this subsection, we will analyze strategy S6. A GDH instance cannot be embedded in the long-term private key in Strategy S6.

- **Setup:** ${Setup}^{list}$ is an initially empty list, and $(ID_{i},\left(d_{i},R_{i}\right),$$PK_{i})$ is needed in this phase. CH creates the system parameters and all parties’ long-term private keys.
  – CH sets *V* as the system public key $P_{pub}$ and exposes $\langle E/F_{p},G,q,P,P_{pub},$$H_{1},H_{2}\rangle$. Thus, CH does not obtain any information about KGC’s master key.
  – For $ID_{a}$, CH sets the long-term private key $\left(⊥,R_{a}\right)$, where $h_{a}\in_{R}Z_{q}^{*}$, $R_{a}=U-h_{a}P_{pub}$. Thus, $PK_{a}=R_{a}+h_{a}P_{pub}=U$.
  – For $ID_{i}\left(i\neq a\right)$, CH sets $\left(s_{i},R_{i}\right)$ as the long-term private key, where $h_{i},s_{i}\in_{R}Z_{q}^{*}$, $R_{i}=s_{i}P-h_{i}P_{pub}$. Thus, $PK_{i}=R_{i}+h_{i}P_{pub}=s_{i}P$.
  – For every participant, CH transfers $\left(ID_{i},R_{i}\right)$ to M, and stores $(ID_{i},$$\left(d_{i},R_{i}\right),PK_{i})$ and $\left(ID_{i},R_{i},h_{i}\right)$ in ${Setup}^{list}$ and ${H_{1}}^{list}$ (described later), respectively.
- **Queries:** CH maintains four lists, ${H_{1}}^{list}$, ${H_{2}}^{list}$, ${Send}^{list}$, and $R^{list}$, which are initially empty and used for recording $H_{1}$, $H_{2}$, Send, and SessionKeyReveal oracles, respectively. CH starts M by answering M’s queries as follows:
  – The five queries $H_{1}\left(ID_{i},R_{i}\right)$, SessionKeyReveal($\prod_{i,j}^{m}$), Test($\prod_{i,j}^{m}$), KGCStaticKeyRevea, and $H_{2}\left(ID_{i},ID_{j},R_{i},R_{j},T_{i},T_{j},K_{1},K_{2}\right)$ are described in the same way as those in Strategy S5.
  – Corrupt($ID_{i}$), EphemeralKeyReveal($\prod_{i,j}^{m}$) and Send($\prod_{i,j}^{m}$,*M*): These three queries are described to be the same as those in Strategy S4.
- **Analysis:** Here, we assume that $\prod_{a,b}^{n}$ is an initiator. If M selects Strategy S6 and $\prod_{a,b}^{n}$ as the test session, then CH continues this simulation. If M successfully performs the forging attack, it should execute $H_{2}$ query on $\left(ID_{a},ID_{b},R_{a},R_{b},T_{a},T_{b},K_{1},K_{2}\right)$, where $K_{1}=\left(DLOG\left(U\right)+C_{1}r_{a,b}^{n}\right)\left(R_{b}+h_{b}V+C_{2}T_{b}\right)$,$K_{2}=\left(DLOG\left(U\right)+C_{3}r_{a,b}^{n}\right)\left(R_{b}+h_{b}V+C_{4}T_{b}\right)$, and $h_{b}=H_{1}\left(ID_{b},R_{b}\right)$. Note that $ID_{a}$’s public key material $R_{a}$ and outgoing message $T_{a}$ are both picked by the challenger CH, and at least one of $ID_{b}$’s public key material $R_{b}$ and outgoing message $T_{b}$ is chosen by M.
  By the forking lemma [27], CH replays M with the same input and tossing coins. Here, CH only changes the query results of $H_{1}\left(ID_{b},R_{b}\right)$, i.e., CH sets $H_{1}\left(ID_{b},R_{b}\right)$ to $\bar{h_{b}}$, where $\bar{h_{b}}\in Z_{q}^{*}$ and $\bar{h_{b}}\neq h_{b}$. Then, if M succeeds, it should perform a query on $H_{2}$ with $\left(ID_{a},ID_{b},R_{a},R_{b},T_{a},T_{b},\bar{K_{1}},\bar{K_{2}}\right)$, where $\bar{K_{1}}=\left(DLOG\left(U\right)+C_{1}r_{a,b}^{n}\right)\left(R_{b}+\bar{h_{b}}V+C_{2}T_{b}\right)$, $\bar{K_{2}}=\left(DLOG\left(U\right)+C_{3}r_{a,b}^{n}\right)$$\left(R_{b}+\bar{h_{b}}V+C_{4}T_{b}\right)$.
  Here, CH receives the item in ${H_{2}}^{list}$, and outputs GDH$\left(U,V\right)={\left(h_{b}-\bar{h_{b}}\right)}^{-1}\left(K_{1}-\bar{K_{1}}\right)-C_{1}r_{a,b}^{n}V$ using the knowledge of $r_{a,b}^{n}$. Note that as $\bar{h_{b}}\neq h_{b}$, the solution of GDH(U,V) is correct. Let λ be a factor from the forking lemma in Strategy S6. CH’s success probability is at least
  $$Adv_{S}^{GDH}\left(k\right)>=\frac{\lambda Adv_{M}^{F}\left(k\right)}{6n_{h}{\left(k\right)}^{2}n_{p}{\left(k\right)}^{2}n_{s}\left(k\right)}.$$
  As $Adv_{M}^{F}\left(k\right)$ is non-negligible, $Adv_{S}^{GDH}\left(k\right)$ can also be seen as non-negligible. Now, it derives the contradiction of the GDH assumption.

The former formal security proof has proven that the proposed ID-AKA protocol $\sum_{C_{1},C_{2},C_{3},C_{4}}$ is secure against some comment attacks of guessing attacks, key replication attacks, and forging attacks. Its security can be reduced to the hardness of GDH assumption over the elliptic curve group in the eCK model.

## 6. More Efficient Instantiations

As $C_{i}\left(i=1,2,3,4\right)\in Z_{q}^{*}$, our construction needs six scalar multiplications (here, we ignore less time-consuming point additions and general hash function outputs), which is a bit higher than the NCL-16-II protocol [27] at the same security level. However, the NCL-16-II protocol is only a special protocol, while our construction will result in different special protocols with different $C_{i}$ values, for example, protocol $\sum_{1,1,-1,-1}$ and protocol $\sum_{1,1,H_{1}(R_{A}\left|\right|R_{B}\left|\right|T_{A}\left|\right|T_{B}),H_{1}(R_{B}\left|\right|R_{A}\left|\right|T_{B}\left|\right|T_{A})}$. How should the values of $C_{1},C_{2},C_{3}$, and $C_{4}$ be chosen in the real execution environment? It would be better to select values that result in more efficient instantiation as different protocols have different computation costs. The following provides some efficient instantiations of our construction.

**Protocol** **1** ($\sum_{1,1,-1,-1}$)**.** *In this protocol, $C_{1}=C_{2}=1,C_{3}=C_{4}=-1$. A computes the shared secrets $K_{AB}^{1}=\left(s_{A}+e_{A}\right)\left(PK_{B}+T_{B}\right)$ and $K_{AB}^{2}=\left(s_{A}-e_{A}\right)\left(PK_{B}-T_{B}\right)$. B compute the shared secrets $K_{BA}^{1}=\left(s_{B}+e_{B}\right)\left(PK_{A}+T_{A}\right)$ and $K_{BA}^{2}=\left(s_{B}-e_{B}\right)\left(PK_{A}-T_{A}\right)$. This protocol reduces two scalar multiplications compared with the general construction.*

**Protocol** **2** ($\sum_{1,-1,-1,1}$)**.** *In this protocol, $C_{1}=C_{4}=1,C_{2}=C_{3}=-1$. A computes the shared secrets $K_{AB}^{1}=\left(s_{A}+e_{A}\right)\left(PK_{B}-T_{B}\right)$ and $K_{AB}^{2}=\left(s_{A}-e_{A}\right)\left(PK_{B}+T_{B}\right)$. B computes the shared secrets $K_{BA}^{1}=\left(s_{B}-e_{B}\right)\left(PK_{A}+T_{A}\right)$ and $K_{BA}^{2}=\left(s_{B}+e_{B}\right)\left(PK_{A}-T_{A}\right)$. This protocol has the same efficiency as $\sum_{1,1,-1,-1}$.*

**Protocol** **3** ($\sum_{1,1,2,2}$)**.** *In this protocol, $C_{1}=C_{2}=1,C_{3}=C_{4}=2$. A computes the shared secrets $K_{AB}^{1}=\left(s_{A}+e_{A}\right)\left(PK_{B}+T_{B}\right)$ and $K_{AB}^{2}=\left(s_{A}+2e_{A}\right)\left(PK_{B}+2T_{B}\right)$. B computes the shared secrets $K_{BA}^{1}=\left(s_{B}+e_{B}\right)\left(PK_{A}+T_{A}\right)$ and $K_{BA}^{2}=\left(s_{B}+2e_{B}\right)\left(PK_{A}+2T_{A}\right)$. This protocol only adds a point addition operation compared with the protocol of $\sum_{1,1,-1,-1}$.*

## 7. Performance and Comparison

This section presents the efficiency and security comparison between our Protocols 1 and 2 with other competitive ID-AKA protocols. Note that only the HC protocols [13] were pairings-based ID-AKA protocols, the other ID-AKA protocols [21,22,23,24,25,26,27,28] and ours were all pairing-free.

### 7.1. Comparison of Computation Overheads

To evaluate the computational overhead, Table 2 lists the same execution time of different cryptographic operations, reported in [40]. The execution time was calculated using the MIRACL library on a Samsung Galaxy S5 smartphone, equipped with a 2.5 GHz ARM Krait processor with 2GB RAM memory running the Android 4.4.2 operating system.

**Figure 6:**
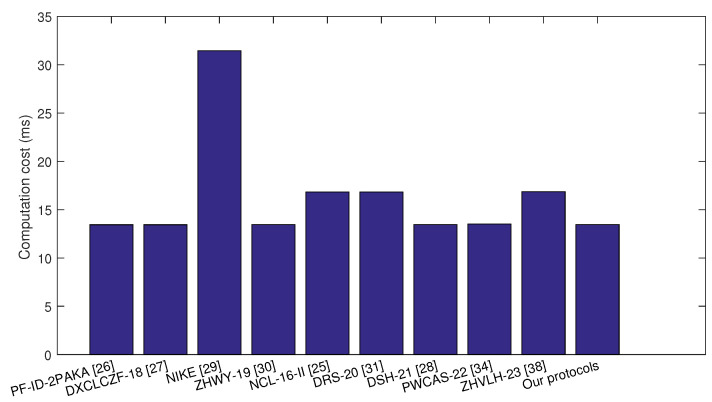
Comparison of computation overheads.

Next, the total execution times of these two protocols and the competitive ID-AKA protocols [13,21,22,23,24,25,26,27,28] were computed, which are shown in Table 3. In our Protocols 1 and 2, to agree on a session key, each party needed to compute four ECC-based scalar multiplications, three ECC-based point additions, and two general hash function outputs. Therefore, the total computation time at each party was about $4T_{esm}+3T_{epa}+2T_{h}\approx 13.454$ ms. Similarly, the communication costs of protocols in [13,21,24,25,26,27,28] were computed. In protocols ZHWY-19 [23] and NIKE [22], two parties had unbalanced computation costs, i.e., one party had a lower computation cost than the other party. Here, we adopted the lower computation cost of one party. According to Table 2, our Protocols 1 and 2 were nearly 80% of protocols NCL-16-II [27] and CKD [24], 100% of protocols [25] and [21,23,28], 72% of protocol XW [26], and 8% of the HC protocol [13] with relation to the computation cost. That is to say, our Protocols 1 and 2 almost had the lowest computation cost. The comparison results are shown in Figure 6.

**Figure 7:**
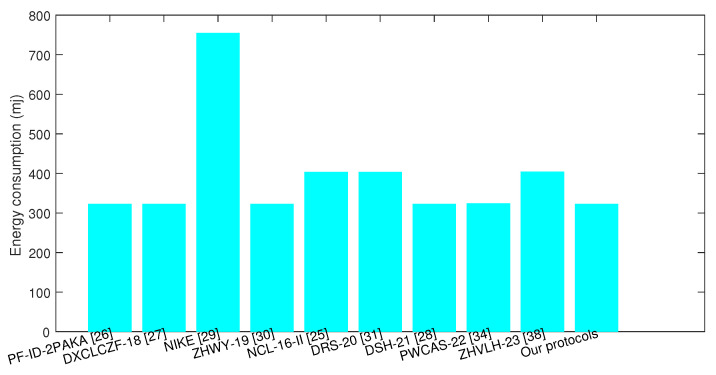
Comparison of energy consumption.

Energy consumption is one essential item for IoT communication, and the energy consumption of ID-AKA protocol decides the energy efficiency of IoT communication as it is executed by the IoT device. As shown in the former comparative computation overheads in Table 3, the computation costs were calculated. To compute the energy consumption of these key agreement algorithms, the IoT devices equipped with 3.0 V and 8.0 mA were selected. This parameter was set according to the power level of MICA 2 [41]. For the proposed ID-AKA protocol, the energy consumption was 3.0 ∗ 8.0 ∗ 13.454 = 322.896 mj, and the comparison results with similar protocols are shown in Figure 7. Therefore, the low computation overheads led to low energy consumption, and the proposed ID-AKA protocol had more advantages than similar protocols in relation to the costs of computation and energy.

### 7.2. Comparison of Communication Overheads

Let $|G_{1}|$, $|G_{2}|$, |G|, and $|Z_{q}^{*}|$ represent elements sizes of $G_{1}$, $G_{2}$, G, and $Z_{q}^{*}$, respectively. Furthermore, assume |ID| and |H| represent the length of an identifier and a general hash output, respectively. Considering the Ate pairing and elliptic curves, $|G_{1}|$, $|G_{2}|$, |G|, $|Z_{q}^{*}|$, and |H| are 1024, 1024, 320, 160, and 160 bits, respectively. We assumed |ID| is 32 bits in length.

Table 4 demonstrates the communication cost comparison of the key agreement phase. Note that in our Protocol 1 (Protocol 2), party *A* sends $\left\{ID_{A},R_{A},T_{A}\right\}$ to party *B*, where $R_{A},T_{A}\in G$ and $ID_{A}$ is the identity of *A*. Party *B* symmetrically sends $\left\{ID_{B},R_{B},T_{B}\right\}$ to party *A*, where $R_{B},T_{B}\in G$ and $ID_{B}$ is the identity of *B*. Therefore, the communication cost of our protocol 1 (protocol 2) is 2∗(|ID|+2|G|)=2∗(32+2∗320)=1344 bits. The results show that Protocols 1 and 2 have the lowest communication cost.

### 7.3. Security Comparisons

As shown in Table 1, some related ID-AKA protocols can capture other security attributes, including known key security, no key control, resistance to basic impersonation attacks, replay attack resilience, resistance to man-in-the-middle attacks, and unknown key share resilience. But, for the proposed IA-AKA protocols, we did not consider explicit mutual authentication, as it can be easily achieved for all one-round protocols [13,21,24,25,26,27,28] by adding a key confirmation. Here, the protocol PWCAS-22 [33] is based on physical unclonable function (PUF), and ZHVLH-23 [37] is based on pseudo-random permutation (PRF). The HC protocol [13], the NCL-16-II protocol [27], the DRS-20 protocol [30], and our protocols are provably secure in the eCK security model. But events in the security proof of NCL-16-II [27] are not complementary, which mismatches the freshness definition. Table 3 shows that our Protocols 1 and 2 can reach the best computation efficiency.

Compared with similar ID-AKA protocols, the proposed Protocols 1 and 2 presented the lowest computation and communication overheads, which could improve IoT communication efficiency in IoT applications. Meanwhile, with the increase in the number of devices, these ID-AKA protocols could also maintain high efficiency, as the key agreement process was executed between two different IoT users. The key agreement between the two parties was less affected by the number of devices in IoT applications and only affected by the hardware, software, and communication protocol in the public internet environment. Although the key agreement times will increase more, this can be ignored with the increasing IoT computation ability.

## 8. Conclusions

This paper first reviews several ID-AKA protocols without pairings in terms of security and efficiency. We carefully studied them and pointed out the security weaknesses against the ephemeral key reveal attack, key compromise impersonation attack, and launch collusion attack. We also proposed a family of ID-AKA protocols without pairings and proven the security in the eCK security model, a widely accepted security model for AKA protocols. Six strategy analyses were provided, and these ID-AKA protocols were proven to be secure in the eCK model based on the GDH assumption over the elliptic curve group. Then, the instantiations, performance and comparison were presented, and the results show that the proposed ID-AKA protocols were more efficient than other protocols in similar literature. In addition, these ID-AKA protocols no only improved communication security and efficiency in IoT applications, but also saved energy consumption for the communication process.

In the future, with the increasing amount of IoT devices, some security issues of identity authentication, data fine-grained access control, and user privacy protection should still be taken into consideration. Especially with the development of quantum computers and quantum computation, the anti-quantum attack security ID-AKA protocol will be a hot research direction. Meanwhile, many customized ID-AKA schemes should be designed to meet the special requirements of future IoT applications.

## Figures and Tables

**Table 1:** Security comparisons.

Protocols	Security Model	wPFS	KCIR	ESRR	MSS	AS
CKD [24]	mBR*	Yes	Yes	No	Yes	GDH
FG-I [25]	CK	Yes	Yes	No	Yes	GDH
XW [26]	CK	Yes	Yes	No	Yes	GDH
PF-ID-2PAKA [21]	eCK* (flawed)	Yes	Yes	No	Yes	GDH
DXCLCZF-18 [28]	eCK (flawed)	Yes	No	No	Yes	GDH
ZHWY-19 [23]	mBR* (flawed)	No	No	No	Yes	GDH
NIKE [22]	– (flawed)	Yes	No	Yes	No	–
NCL-16-II [27]	eCK@	Yes	Yes	Yes	Yes	GDH
DRS-20 [30]	eCK	Yes	Yes	Yes	Yes	GDH
DSH-21 [29]	eCK*	Yes	Yes	Yes	Yes	GDH
PWCAS-22 [33]	eCK*	Yes	Yes	Yes	Yes	PUF
ZHVLH-23 [37]	eCK*	Yes	Yes	Yes	Yes	PRF

eCK* and mBR* are without the case where an active adversary may alter all public key materials not only the temporary public key. “–” denotes that there is no formal security proof for the protocol. eCK@ denotes that events in the proof are not complementary.

**Table 2:** Execution time on a Samsung Galaxy S5.

Notation	Explanation (The Execution Time of)	Time (ms)
$T_{p}$	A bilinear pairing *e*: $G_{1}\times G_{1}\to G_{2}$	32.713
$T_{psm}$	A pairing-based scalar multiplication in $G_{1}$	13.405
$T_{ppa}$	A pairing-based point addition in $G_{1}$	0.056
$T_{pexp}$	An exponentiation operation in $G_{2}$	2.249
$T_{mtph}$	A hash-to-point in $G_{1}$	33.582
$T_{esm}$	An ECC-based scalar multiplication in G	3.350
$T_{epa}$	An ECC-based point addition in G	0.014
$T_{emtph}$	A hash-to-point in G	8.250
$T_{h}$	A general hash function	0.006

**Table 3:** Comparative computation overheads.

Protocols	Computations	Computation Cost (ms)	Energy Consumption (mj)
PF-ID-2PAKA [21]	$4T_{esm}+2T_{epa}+2T_{h}$	13.44	322.56
DXCLCZF-18 [28]	$4T_{esm}+2T_{epa}+2T_{h}$	13.44	322.56
NIKE [22]	$2T_{esm}+3T_{emtph}$	31.45	754.8
ZHWY-19 [23]	$4T_{esm}+3T_{epa}+3T_{h}$	13.46	323.04
NCL-16-II [27]	$5T_{esm}+4T_{epa}+3T_{h}$	16.824	403.776
DRS-20 [30]	$5T_{esm}+3T_{epa}+5T_{h}$	16.822	403.728
DSH-21 [29]	$4T_{esm}+3T_{epa}+3T_{h}$	13.460	323.04
PWCAS-22 [33]	$4T_{esm}+6T_{epa}+5T_{h}$	13.514	324.336
ZHVLH-23 [37]	$5T_{esm}+T_{epa}+16T_{h}$	16.860	404.64
Our protocols	$4T_{esm}+3T_{epa}+2T_{h}$	13.454	322.896

**Table 4:** Comparative communication overheads.

Protocols	Messages No.	Communication Cost	Cost (bits)
PF-ID-2PAKA [21]	2	2|ID|+4|G|	1344
DXCLCZF-18 [28]	2	2|ID|+6|G|	1984
ZHWY-19 [23]	3	$6|G|+|Z_{q}^{*}\left|+2|H\right|$	2400
NIKE [22]	3	2|ID|+5|G|	1664
NCL-16-II [27]	2	2|ID|+6|G|	1984
DRS-20 [30]	2	2|ID|+4|G|+|H|	1504
DSH-21 [29]	2	2|ID|+4|G|	1344
PWCAS-22 [33]	2	2|ID|+4|G|+2|H|	1664
ZHVLH-23 [37]	2	3∗(2|ID|+|G|+|H|)	1632
Our protocols	2	2|ID|+4|G|	1344

## Data Availability

Data are contained within the article.
